# Antidiabetic Effect of Fenugreek Seed Powder Solution (*Trigonella foenum-graecum L*.) on Hyperlipidemia in Diabetic Patients

**DOI:** 10.1155/2019/8507453

**Published:** 2019-09-05

**Authors:** Genet Alem Geberemeskel, Yared Godefa Debebe, Nigisty Abraha Nguse

**Affiliations:** Department of Medical Physiology, Institute of Bio-Medical Sciences, College of Health Sciences, Mekelle University, Mekelle, Ethiopia

## Abstract

**Background:**

Many drugs are commercially available for use in the management of diabetes. However, their side effects and high costs underscore the need for herbal alternative drugs. *Trigonella foenum-graecum* is one of the medicinal plants which are important in the management of diabetes mellitus. This study investigated the effect of *Trigonella foenum-graecum* seed powder solution on the lipid profile of newly diagnosed type II diabetic patients.

**Methods:**

A total of *N* = 114 newly diagnosed type II diabetic patients without any significant diabetes complication were selected. They were grouped into two groups: the treatment group (*n* = 57) consumed 25 g *Trigonella foenum-graecum* seed powder solution orally twice a day for one month and the second group is the control (*n* = 57) which receives metformin. Blood sample was collected from each participant by a medical technologist/technician before and after the study. Lipid profile was analyzed by using Mindray BS 200E fully automated clinical chemistry analyzer.

**Result:**

By the end of the intervention period, the treatment group showed significantly lower total cholesterol level by 13.6% as compared with the baseline level (219.1 ± 35.51 vs. 189.29 ± 29.06, *P* < 0.001) and the control group (189.29 ± 29.06 vs. 208.2 ± 40.2, *P* < 0.001); triglyceride level also reduced by 23.53% compared with the baseline level (256.1 ± 15.4 vs. 195.8 ± 82.95, *P* < 0.001) and compared with the control group (195.8 ± 82.95 vs. 244.1 ± 96.9, *P* < 0.05); and low-density lipoprotein cholesterol level also reduced by 23.4% as compared to the baseline level (137.9 ± 26.9 vs. 105.6 ± 24.2, *P* < 0.001) and the control group (between groups) (105.6 ± 24.2 vs. 144.1 ± 23.3, *P* < 0.001), but the treatment group showed significantly increased high-density lipoprotein cholesterol level by 21.7% as compared to the baseline level, within group (37.8 ± 1.51 vs. 48.3 ± 11.9, *P* < 0.001), and the control group, between groups (48.3 ± 11.9 vs. 36.01 ± 9.5, *P* < 0.001). However, lipid profile levels in the control group were not significantly changed.

**Conclusion:**

The present study showed that the administration of *Trigonella foenum-graecum* seed powder solution had pronounced effects in improving lipid metabolism in type II diabetic patients with no adverse effects. Therefore, *Trigonella foenum-graecum* seed may provide new alternatives for the clinical management of type II diabetes.

## 1. Background

Diabetes mellitus (DM) is a metabolic disorder characterized by chronic hyperglycemia either because the pancreas does not produce enough insulin or the peripheral target tissues are unable to respond to the normal concentration of insulin [1, 2]. It is a major cause of morbidity and mortality with an increasing prevalence and the fastest growing disease worldwide [3, 4]. The WHO estimates a prevalence of 347 million people with diabetes and an estimated 4.6 million deaths each year [5]. The prevalence is expected to double in 2030, and the greater proportion of this increase would be in the low- to middle-income countries of Asia, Africa, and South America [4].

The most frequent form is type 2 diabetes which represents more than 90-95% of the cases [6]. This form was previously referred to as non-insulin-dependent DM (NIDDM) or “adult-onset diabetes.” In most cases, the onset of type 2 diabetes occurs after 30 years, often between the ages of 40 and 60 years. In recent years, however, there has been a steady increase in the number of younger individuals, some less than 20 years old [7, 8].

Dyslipidemia is common in type 2 diabetes, as both insulin deficiency and resistance affect enzymes and pathways of lipid metabolism [9]. Dyslipidemia is characterized by hypercholesterolemia, hypertriglyceridemia, increased levels of low-density lipoprotein cholesterol (LDL-C), and decreased HDL-C [1]. Disturbances of lipid metabolism appear to be an early event in the development of type 2 diabetes, potentially preceding the disease by several years [10].

The current approaches for the treatment of diabetes mostly depend on insulin injection and oral antidiabetic agents [11–15]. Modern drugs which are used for the treatment of type 2 diabetes control the blood glucose and lipid profile level as long as they are regularly administered [13, 16, 17].

Despite their effectiveness, they have unwanted side effects and toxicity, including nausea, vomiting, hematological and dermatological reactions, obstructive jaundice, hyponatremia, and intolerance of alcohol and weight gain [18]. In addition to the harmful side effects, limitation of synthetic drugs includes, shortage, lack of equitable distribution, unaffordability, and less acceptable by the patients. Diabetic patients are at risk of chronic morbidity and premature mortality despite the assiduous use of the available drugs [19–21]. Due to these several side effects, there is a strong desire to use herbs or plants for treatment, due to less side effects, less toxic, and easier consumption or availability as well as low cost as compared to synthetic counterparts [22, 23]. Recently, many medicinal plants have demonstrated the potential for the treatment of type 2 diabetes and its complications. One such promising plant is *Trigonella foenum-graecum L*. [24, 25].

*Trigonella foenum-graecum* is a useful medicinal plant belonging to family Fabaceae [24]. It is an annually grown herb which is cultivated throughout the world including Ethiopia. *Trigonella foenum-graecum* is suitable for areas with moderate or low rainfall. It is an erect plant with a height of 30–60 cm, with compound pinnate trifoliate leaves, auxiliary white to yellowish flowers, and 3–15 cm long thin pointed beaked pods, which contain 10–20 oblong greenish-brown seeds [23, 25]. The seeds are used as spices worldwide, whereas the leaves are used as green leafy vegetables in a diet. *Trigonella foenum-graecum* seeds are bitter to taste and are known for a long time for their medicinal qualities [21, 23, 24]. Ancient literature, religious scripture, travel records, and anecdotes from different continents and from different periods of human history record a wide variety of medicinal properties associated with *Trigonella foenum-graecum*. Medicinal uses vary from wound healing to bust enhancement and from promotion of lactation in weaning mothers to its use as a sex stimulant or aphrodisiac [13, 20, 22]. The medicinal plants provide a useful source of oral antihyperglycemic bioactive compounds for the development of new pharmaceutical clues as well as a good source of dietary supplement to existing therapies. Therefore, the main purpose of this study was to investigate the effect of *Trigonella foenum-graecum* seed powder solution on the lipid profile in newly diagnosed type II diabetic patients (patients which had abnormal blood glucose levels showed fasting blood glucose levels of ≥150 mg/dL and abnormal lipid profile but did not start any treatment yet).

## 2. Methods

### 2.1. Study Area

The study was conducted at Ayder Referral Hospital and Mekelle General Hospital located in Mekelle city (39°29′E and 13°30′N), the capital city of Tigray Regional State.

### 2.2. Study Design

Experimental study using quantitative methods was conducted to evaluate the effect of *Trigonella foenum-graecum* seed powder solution in newly diagnosed type II diabetic patients. This study was conducted over a period from May, July 2, 2015, to January 31, 2016.

### 2.3. Eligibility Criteria

#### 2.3.1. Inclusion Criteria

All newly diagnosed type II diabetic patients in Ayder Referral Hospital and Mekelle General Hospital during the study period, who do not fall under the exclusion criteria, were included in the study.

#### 2.3.2. Exclusion Criteria

- Patients who are not willing to participate in the study and unable to give informed consent
- Patients with any diabetic complications
- Patients who are currently taking antidiabetic medications
- Patients who are pregnant and lactating
- Patients on lipid-lowering therapy
- Human immunodeficiency virus (HIV) infection

### 2.4. Sample Size Determination

The sample size was calculated using a double population proportion formula.

By assumption, significance level is equal to 95%, power is equal to 80%, the type of test is two‐sided, and *Z*_*α*/2_ is the critical value at 95% confidence level of certainty (1.96).

The actual sample size for comparison of two proportions (two-sided) is as follows:

(1) $$\begin{array}{c} n=\frac{{\left[z_{\alpha /2}+z_{\beta }\right]}^{2}\times \left[\pi_{1}\left(1-\pi_{1}\right)+\pi_{2}\left(1-\pi_{2}\right)\right]}{{\left(\pi_{1}-\pi_{2}\right)}^{2}}, \end{array}$$

where *n* is the sample size required in each group (double this for total sample), *π*_1_ is the first proportion (proportion of outcome on controls) which is equal to 0.50, *π*_2_ is the second proportion (proportion of outcome on treatment group) which is equal to 0.2, and *π*_1_ − *π*_2_ is the size of difference of clinical importance which is equal to 0.3. *z*_*α*/2_ = 1.96, the value of the standard normal distribution corresponding to a significance level of 1.96 for a 2-sided test at the 0.05 level. *z*_*β*_ = 0.84, the value of the standard normal distribution corresponding to the desired level of power (0.84 for a power of 80%).

Inserting the required information into the formula gives

(2) $$\begin{array}{c} n=\frac{\left(1.96+0.84\right)2*\left(\left(0.5*0.5\right)+\left(0.2*0.8\right)\right)}{\left(0.5-0.2\right)2}=35.7. \end{array}$$

This value (35) is required in each of the trials of the two groups (35 for the treatment and 35 for the control).

Taking into consideration 10% for the nonresponse rate and 1.5 for the design effect of the sample size, *N* for each group was equal to 57; therefore, a total of *N* = 114 patients were recruited to participate in both groups.

### 2.5. Sampling Technique

Patients who satisfied the eligibility criteria were randomized into treatment and control groups. The treatment group received 25 mg of *Trigonella foenum-graecum* seed powder solution twice daily for one month, while controls received none.

### 2.6. Response Rate

**Figure 1:**
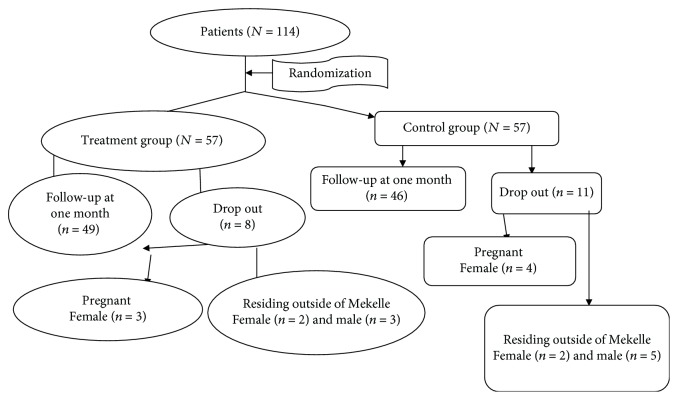
Flow chart describing the number of participants in the treatment and control groups who participated in the study. Dependent variable: primary end points: TC, TG, HDL-C, and LDL-C. Independent variable: the intervention variable was *Trigonella foenum-graecum* seed powder solution. Other covariants include age, sex, marital status, level of education, and occupational status.

A total of *N* = 114 patients were recruited for the study. Out of these, *N* = 95 completed the study, *n* = 49 in the treatment group and *n* = 46 in the control group, but some dropped out of the study: *n* = 4 patients in the first two weeks, *n* = 6 patients in the second two weeks, and *n* = 9 patients at the end of the treatment for reasons unrelated to the use of *Trigonella foenum-graecum* seed powder solution (Figure 1).

### 2.7. Preparation of *Trigonella foenum-graecum* Seed Powder Solution

Good quality *Trigonella foenum-graecum* seeds were purchased from the local market of Mekelle city. The seeds were winnowed, washed with clean water, and dried in sunlight. After drying, the seeds were grinded with an electric grinder and passed through a 1 mm mesh sieve. A dose of 25 mg seed powder was weighed by a digital electronic balance and packed in a clean plastic container. Then, the 25 mg of the *Trigonella foenum-graecum* seed powder was soaked in 1 L of water for 10 hours. After 10 hours, the solution was separated into filtrate and residue. The filtrate obtained from this procedure was removed, and the residue was given to the treatment group as a single dose of treatment.

### 2.8. Treatment and Control Groups

Both the treatment group and the control group of the study had abnormal blood glucose levels, showed fasting blood glucose levels of ≥150 mg/dL, and had abnormal lipid profile. The treatment group took 25 mg of *Trigonella foenum-graecum* seed powder solution and the control group received neither *Trigonella foenum-graecum* nor antidiabetic agent, but all the participants of the treatment and control groups were recommended to eat proper diet and do exercise by their doctor.

### 2.9. Data Collection and Measurable Methods

Preliminary data was collected at the baseline by preparing a standardized questionnaire that contains information on the sociodemographic factors and behavioral and clinical characteristics related to the disease.

Measurements were made at baseline (before treatment) and at the end of intervention. Blood was collected at 8:00 am from the antecubital vein, while the patients were in the recumbent position after an overnight fasting, from both treatment and control groups by a medical laboratory technologist into a serum separator test tube (SST). After 15 minutes, the blood sample was centrifuged at 3000 RPM for five minutes. The serum was transferred to sterile tubes and stored at 2-8°C until analysis. Then, TC, TG, HDL-C, and LDL-C levels were analyzed by using a BS 200E chemistry analyzer. The measured values of lipid concentrations were compared with baseline and the control group.

### 2.10. Data Analysis

Data was analyzed using statistical package for social sciences (SPSS) version 20 for Windows software. Statistical analysis was carried out using Student's paired and independent *t*-test. Paired sample *t*-test was used to determine the significance within the groups, and independent sample *t*-test was used to determine the significant difference between the mean of the lipid profile of the treatment and control groups. Results of the study are expressed as mean ± SD, and statistical bar graphs were drawn with Microsoft Excel 2007. *P* < 0.05 is considered as statistically significant.

### 2.11. Ethical Consideration

Appropriate ethical approval was granted from Mekelle University College of Health Sciences, Health Research Ethics Review Committee (HRERC) of Ayder Referral and Comprehensive Specialized Hospital, registration number ERC 0647/2015, and I have obtained written informed consent from each study participant.

## 3. Results

The sociodemographic and behavioral and clinical characteristics of the study participants are shown in Tables 1 and 2, respectively.

### 3.1. Baseline Blood Glucose Level and Lipid Profile of the Study Participants

As can be seen in Table 3 and Table 4, at the beginning of the study, both the treatment and control groups had abnormal fasting blood glucose levels (≥180 mg/dL) and abnormal lipid profile (TC, TG, HDL-C, and LDL-C).

### 3.2. Effect of *Trigonella foenum-graecum* Seed Powder Solution on Blood Lipid Profile

#### 3.2.1. The Effects of *Trigonella foenum-graecum* Seed Powder Solution on Total Cholesterol (TC)

**Figure 2:**
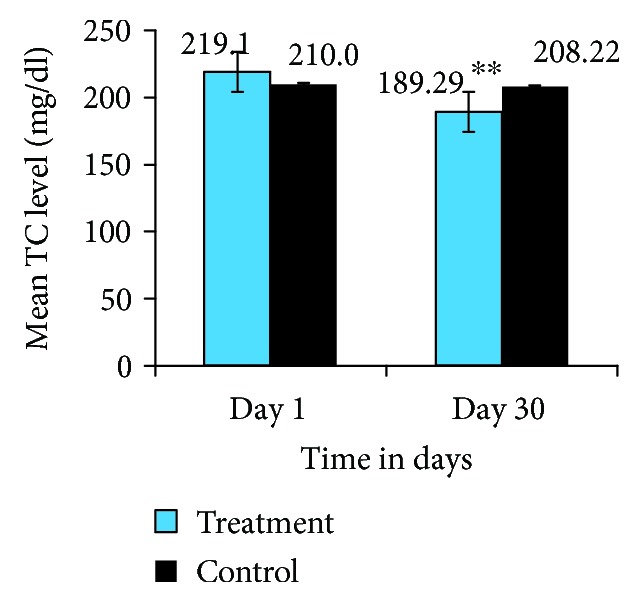
TC level of the treatment and control groups on day 1 and day 30. ^∗∗^Indicates significant differences (*P* value ≤ 0.001), day 1 vs. day 30 treatment group. ^∗∗^Indicates significant difference (*P* value ≤ 0.001), treatment vs. control on day 30.

The treatment group, administered with 25 mg *Trigonella foenum-graecum* seed powder solution for 30 consecutive days, showed a significant reduction of 13.6% in serum TC level as compared with the baseline TC level, within group (219.1 ± 35.51 vs. 189.29 ± 29.06, *P* < 0.001), and the TC level of the control group, between groups (189.29 ± 29.06 vs. 208.2 ± 40.2, *P* < 0.001), on day 30. However, there was no significant difference in TC level in the control group (210.02 ± 41.18 vs. 208.2 ± 40.2, *P* < 0.22), on day 30 (Figure 2).

#### 3.2.2. The Effects of *Trigonella foenum-graecum* Seed Powder Solution on Triglyceride (TG)

**Figure 3:**
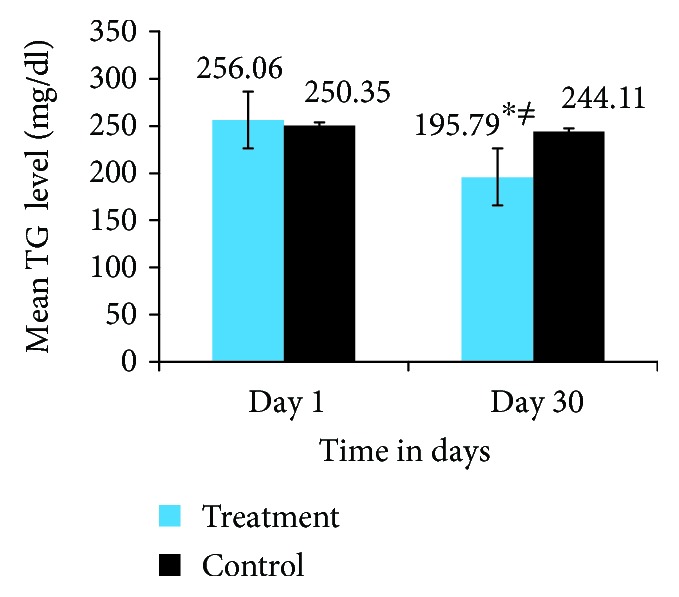
TG level of the treatment and control groups on day 1 and day 30. The results are expressed as mean ± SD. ^∗∗^Indicates significant differences (P value ≤ 0.001), day 1 vs. day 30 treatment group. *^≠^*Indicates significant differences (*P* value ≤ 0.05), treatment group vs. control group on day 30.

The data shows that the treatment group receiving *Trigonella foenum-graecum* seed powder solution for 30 consecutive days had a statistically significant decrease of 23.53% in serum TG level compared with baseline TG level (256.1 ± 15.4 vs. 195.8 ± 82.95, *P* < 0.001) and compared with the TG level of the control group, between groups (195.8 ± 82.95 vs. 244.1 ± 96.9, *P* < 0.05), on day 30, but the control group had no significant difference in TG level (250.35 ± 96.9 vs. 244.1 ± 96.9, *P* < 0.21) on day 30 (Figure 3).

#### 3.2.3. The Effects of *Trigonella foenum-graecum* Seed Powder Solution on High-Density Lipoprotein Cholesterol (HDL-C)

**Figure 4:**
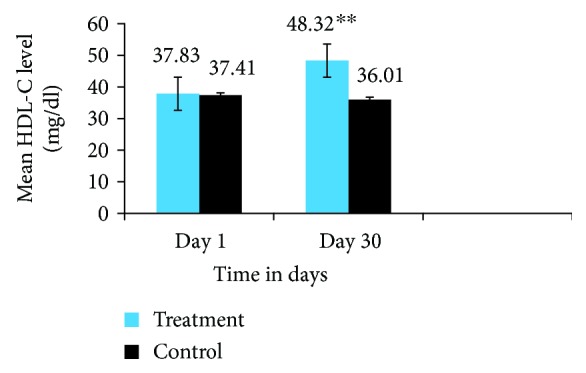
HDL-C level of the treatment and control groups on day 1 and day 30. The results are expressed as mean ± SD. ^∗∗^Indicates significant differences (*P* value ≤ 0.001), day 1 vs. day 30 treatment group. ^∗∗^Indicates significant differences (*P* value ≤ 0.001), treatment group vs. control group on day30.

Figure 4 shows that the administration of *Trigonella foenum-graecum* seed powder solution for 30 consecutive days significantly increased the serum HDL-C level of the treatment group by 21.7% as compared to the baseline HDL-C level, within group (37.8 ± 1.51 vs. 48.3 ± 11.9, *P* < 0.001), and the HDL-C level of the control group, between groups (48.3 ± 11.9 vs. 36.01 ± 9.5, *P* < 0.001), on the 30^th^ day. The control group had no significant difference in serum HDL-C (37.41 ± 9.2 vs. 36.01 ± 9.5, *P* < 0.15).

#### 3.2.4. The Effects of *Trigonella foenum-graecum* Seed Powder Solution on Low-Density Lipoprotein Cholesterol (LDL-C)

**Figure 5:**
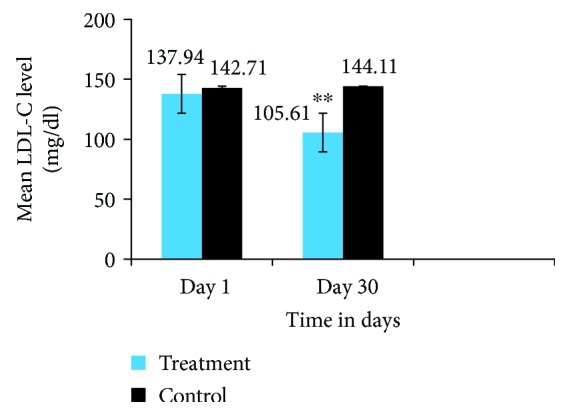
LDL-C level of the treatment and control groups on day 1 and day 30. The results are expressed as mean ± SD. ^∗∗^Indicates significant differences (*P* value ≤ 0.001), day 1 vs. day 30 treatment group. ^∗∗^Indicates significant differences (*P* value ≤ 0.001), treatment vs. control on day 30.

As can be seen from Figure 5, the treatment group receiving *Trigonella foenum-graecum* seed powder solution for 30 consecutive days had a significant reduction by 23.4% in serum LDL-C level as compared to the baseline LDL-C level, within group (137.9 ± 26.9 vs. 105.6 ± 24.2, *P* < 0.001), and the LDL-C level of the control group, between groups (105.6 ± 24.2 vs. 144.1 ± 23.3, *P* < 0.001), on day 30. However, the control groups had insignificant change in serum LDL-C level on day 30 (142.71 ± 23.8 vs. 144.1 ± 23.3, *P* < 0.333).

## 4. Discussion

Diabetes mellitus has high prevalence, morbidity, and mortality globally. Management of type 2 diabetes is difficult with synthetic drugs as they cause many side effects and have some limitations. Thus, as an alternative, there is an immense interest in medicinal plants for managing type 2 diabetes with indigenous, inexpensive, food-based treatment. Scientists have started looking into herbal extracts to observe their effective and protective role in diabetic animal models and humans.

One of such herbal plants is *Trigonella foenum-graecum*. The present work demonstrates a significant role of *Trigonella foenum-graecum* seed powder solution in improving dyslipidemia in newly diagnosed type II diabetic patients.

In this study, dyslipidemia was significantly improved in the treatment group by the administration of 25 mg *Trigonella foenum-graecum* seed powder solution for 30 consecutive days. Significantly reduced TC, TG, and LDL-C (*P* < 0.001) and increased HDL-C (*P* < 0.001) were observed as compared with their baseline lipid profile level (within group) and when we compared with the control group (between groups) (Figures 2–5).

In concordance with the result of our studies done on the effect of fenugreek seeds on glycemia and dyslipidemia in patients with type 2 diabetes mellitus after 8 weeks of treatment, there was a significant improvement in TC (350 ± 20.6 to 176 ± 17.2; *P* < 0.0001), TGs (280 ± 18.2 to 132 ± 16.8; *P* < 0.0001), LDL-C (220 ± 21.4 to 96 ± 14.2; *P* < 0.0001), and HDL-C (27.0 ± 13.4 to 58 ± 32.2; *P* < 0.0002) by Prasanna [23], Mitra and Bhattacharya [26], Lu et al. [27], and Kumar et al. [4]. Thus, the result suggests that *Trigonella foenum-graecum* seed powder solution has a potential antidyslipidemia effect although the mechanism of action is not well defined. But several hypotheses have been put forward in this respect. In a recent study, the reductions of TC, TG, and LDL-C levels and increase in HDL-C level by *Trigonella foenum-graecum* seed powder solution might be hypostasized due to crude fiber and saponin content in *Trigonella foenum-graecum* seed and estrogenic constituent, indirectly increasing thyroid hormone [28]. *Trigonella foenum-graecum* seed increased fecal bile acid and cholesterol excretion. This may be secondary to a reaction between the bile acids and fenugreek-derived saponins causing the formation of micelles too large for the digestive tract to absorb [28]. *Trigonella foenum-graecum* seed powder solution may delay the absorption of glucose and fatty acids, thus providing less substrate for the synthesis of triglycerides [29].

On the other hand, results of the current study are in contrast with previous studies conducted by Kassaian et al. [30] and Gaddam et al. [31] who reported no significant effect of *Trigonella foenum-graecum* seed powder on TC, HDL-C, and LDL-C levels between treatment and control groups. These discrepancies might be possibly due to methodological issues such as differences in the method of preparation, dose, and species of *Trigonella foenum-graecum* seed given.

## 5. Conclusions

The present study showed that the *Trigonella foenum-graecum* seed powder solution taken by newly diagnosed type II diabetic patients produced a significant reduction in TC, TG, and LDL-C levels and increase in HDL-C level.

This investigation reveals that *Trigonella foenum-graecum* seed powder solution is a potent natural food source that has a capacity to control dyslipidemia. In order to provide adequate confirmation, more research including comprehensive chemical and pharmacological investigation should be carried out to isolate and characterize a specific bioactive compound of the *Trigonella foenum-graecum* seed powder, and appropriate elucidation of its mechanism of action needs future study. And also, it needs further comprehensive work to assess at a larger scale and long-term outcomes of *Trigonella foenum-graecum* seed powder solution.

## Figures and Tables

**Table 1:** Sociodemographic characteristics of the study participants.

Variables	Response	Treatment *N* (%)	Control *N* (%)
Sex	Male	27 (55.1%)	27 (58.7%)
	Female	22 (44.9%)	19 (41.3%)

Age	<35	0	2 (4.3%)
	35-44	4 (8.2%)	5 (10.9%)
	45-54	23 (46.9%)	12 (26.1%)
	55-64	15 (30.6%)	16 (34.8%)
	65-74	6 (12.2%)	11 (23.9)%
	≥75	1 (2.0%)	

Residence	Rural	7 (14.3%)	6 (13%)
	Urban	42 (85.7%)	40 (87%)

Job	Government	19 (38.8%)	22 (47.8%)
	Self-employed	13 (26.5%)	11 (23.9%)
	Housewife	11 (22.4%)	9 (19.6%)
	Farmer	6 (12.2%)	4 (8.7%)

**Table 2:** Behavioral and clinical characteristics of the study participants.

Variables	Response	Treatment *N* (%)	Control *N* (%)
Alcohol consumption	No	16 (32.7%)	8 (17.4%)
	Yes	33 (67.3%)	38 (82.6%)

Exercise habit	No	37 (75.5%)	31 (67.4%)
	Yes	12 (24.5%)	15 (32.6%)

Duration of the disease (years)	<1	30 (61.2%)	27 (58.7%)
	1-5	14 (28.6%)	16 (34.8%)
	6-10	5 (10.2%)	3 (6.5%)

Smoking history	Yes	5 (10.2%)	7 (15.2%)
	No	44 (89.8%)	39 (84.8%)

BMI (kg/m^2^)	Normal (18.5-24.9)	18 (36.7%)	22 (47.8%)
	Overweight (25-29.9)	24 (49%)	18 (39.1%)
	Obesity (≥30)	7 (14.35)	6 (13%)

Family history of diabetes	No	36 (73.5%)	30 (65.2%)
	Yes	13 (26.5%)	16 (34.8%)

**Table 3:** Baseline fasting blood glucose level and lipid profile measured on day 1.

Parameters baseline value	Treatment group	Control group
FBG (mg/dL)	184.9 ± 38.9	181.5 ± 16.4
TC (mg/dL)	219.1 ± 35.5	210.02 ± 41.2
TG (mg/dL)	256.1 ± 15.4	250.4 ± 96.9
HDL-C (mg/dL)	37.8 ± 11.5	37.4 ± 9.2
LDL-C (mg/dL)	137.9 ± 26.9	142.7 ± 23.8

Data are expressed as mean ± SD.

**Table 4:** Baseline lipid profile measured on day 1.

Parameters baseline value	Treatment group	Control group
TC (mg/dL)	219.1 ± 35.5	210.02 ± 41.2
TG (mg/dL)	256.1 ± 15.4	250.4 ± 96.9
HDL-C (mg/dL)	37.8 ± 11.5	37.4 ± 9.2
LDL-C (mg/dL)	137.9 ± 26.9	142.7 ± 23.8

Data are expressed as mean ± SD.

## Data Availability

The datasets used and/or analyzed during the current study are available from the corresponding author upon reasonable request.

## Abbreviations

ADA: American Diabetes Association
CI: Confidence interval
DM: Diabetes millets
HDL-C: High-density lipoprotein cholesterol
IDDM: Insulin-dependent diabetes mellitus
IDF: International Diabetes Federation
IRE: Institutional review board
IR: Insulin resistance
LDL-C: Low-density lipoprotein cholesterol
MLT: Medical laboratory technologist
NIDDM: Non-insulin-dependent diabetes mellitus
RPM: Revolution per minute
SEM: Standard error of mean
SPSS: Statistical package for social science
TG: Triglycerides
TLC: Total cholesterol level
VLD-C: Very low-density lipoprotein cholesterol
vs.: Versus
WHO: World Health Organization.
